# Dual function of SF3B2 on chromatin and RNA to regulate transcription in head and neck squamous cell carcinoma

**DOI:** 10.1186/s13578-022-00812-8

**Published:** 2022-06-17

**Authors:** Koji Kitamura, Hidefumi Suzuki, Ryota Abe, Hidenori Inohara, Yasufumi Kaneda, Hidehisa Takahashi, Keisuke Nimura

**Affiliations:** 1 Division of Gene Therapy Science, Department of Genome Biology, Osaka University Graduate School of Medicine, 2-2 Yamada-oka, Suita, Osaka 565-0871 Japan; 2 Department of Otorhinolaryngology-Head and Neck Surgery, Osaka University Graduate School of Medicine, Suita, Osaka 565-0871 Japan; 3 Department of Molecular Biology, Yokohama City University Graduate School of Medical Science, Yokohama, Kanagawa 236-0004 Japan

## Abstract

RNA is spliced concomitantly with transcription and the process is organized by RNA splicing factors, transcriptional regulators, and chromatin regulators. RNA is spliced in close proximity to transcription machinery. Hence, some RNA splicing factors may play a role in transcription. Here, we show that the splicing factor SF3B2 binds to gene regulatory elements and mRNA to modulate transcription and RNA stability in head and neck squamous cell carcinoma cells. High SF3B2 expression leads to poor prognosis in patients with head and neck squamous cell carcinoma and to progression of tumor growth in mice. SF3B2 promotes tumor growth, owing to its involvement in activation of gene expression associated with mitochondrial electron transport and transcription regulatory region DNA binding. SF3B2 is enriched around the promoter element on chromatin and the transcription termination site on RNA. SF3B2 is involved in the regulation of RNA stability. According to the SF3B2-binding profile, SF3B2 regulates RNA polymerase II activity, in addition to regulating RNA splicing. Mechanistically, SF3B2 promotes the binding of structural maintenance of chromosomes 1A and CCCTC-binding factor (CTCF) to the SF3B2-binding genomic regions. SF3B2 also modulates CTCF transcriptional activity. Our findings indicate that SF3B2 has a dual function in both transcription and RNA stability, leading to head and neck squamous cell carcinoma progression.

**Supplementary Information:**

The online version contains supplementary material available at 10.1186/s13578-022-00812-8.

## Introduction

RNA splicing is a critical step in the maturation of mRNA, for removing introns from pre-mRNA. Activities of proteins can be changed by regulating the splicing patterns of protein-coding mRNAs. Cancer cells acquire an advantage in tumor growth and resistance to anti-cancer drugs, owing to splicing variants generated by aberrant expression and mutations in RNA splicing factors [1]. Mutations in RNA splicing genes, including splicing factor 3b subunit 1 (SF3B1), serine and arginine rich splicing factor 2 (SRSF2), U2 small nuclear RNA auxiliary factor 1 (U2AF1), and zinc finger CCCH-type RNA-binding motif and serine/arginine rich 2 (ZRSR2), are often detected in hematopoietic tumors, such as refractory anemia with ringed sideroblasts (RARS) and chronic myelomonocytic leukemia (CMML), while solid tumors have a lower rate of mutations in these RNA splicing factors, except for SF3B1 in uveal melanoma [2]. RNA splicing factor expression levels have been reported to contribute to RNA splicing patterns and gene expression profiles in solid tumors [3, 4].

Splicing factor 3b subunit 2 (SF3B2, also known as SAP145 and SF3b145) is a component of the SF3b complex assembled into the U2 snRNP complex [5]. SF3B2 plays a role in the U2 snRNP complex to splice introns from the pre-mRNA. In addition, SF3B2 directly interacts with the RNA sequence coding cryptic exon and increases the expression of a splicing variant that promotes malignancy in castration-resistant prostate cancer (CRPC) [6]. High SF3B2 expression is associated with poor prognosis, at least in prostate cancer, bladder cancer, acute myeloid leukemia (AML), lung adenocarcinoma, breast cancer, and head and neck squamous cell carcinoma (HNSCC) [6]. Thus, SF3B2 has a distinct role in RNA splicing from the U2 snRNP complex, and its high expression contributes to tumor progression.

RNA splicing is coupled with elongation of transcription. The phosphorylation status of the carboxy-terminal domain of RNA polymerase II (Pol2) is critical for recruiting RNA splicing machinery [7]. Pol2 pauses at the transcription start site (TSS) and the splice sites of the retained exon, suggesting that Pol2 elongation speed is kinetically associated with RNA splicing [8, 9]. Transcription regulating machinery also controls RNA splicing [1, 10]. CCCTC-binding factor (CTCF) and cohesin modulate chromatin architecture and gene expression [11–14]. Binding of CTCF to an exon facilitates the inclusion of the target exon by pausing Pol2 [15]. These results indicate a close relationship between chromatin architectural control and RNA splicing during gene expression and regulation of RNA splicing by transcription-regulating machinery. However, it remains unclear whether RNA splicing factors modulate transcription.

In the present study, we revealed that SF3B2 regulates transcription activity of structural maintenance of chromosomes 1A (SMC1A), a cohesin component, and CTCF, in parallel with RNA splicing regulation. SF3B2 signals were enriched in gene expression regulatory regions on chromatin and near transcription termination sites on RNA. SF3B2 promoted SMC1A loading on the SF3B2-binding genomic elements and was associated with the suppression of CTCF-mediated transcription activation. High SF3B2 expression resulted in the activation of mitochondria-related gene expression. High SF3B2 expression was also associated with poor overall survival in patients with HNSCC and promoted tumor growth in mouse HNSCC xenograft model. Collectively, our findings indicate that SF3B2 has a dual function in gene regulatory elements and RNA, suggesting an SF3B2-mediated feedback loop between transcription and RNA abundance.

## Results

### SF3B2 promotes tumor progression in patients with HNSCC and in mouse xenograft models

High SF3B2 expression promotes tumor progression in CRPC by increasing the expression of androgen receptor splicing variant 7 (AR-V7) in prostate cancer [6]. High SF3B2 expression is also associated with poor overall survival in patients with other cancers.

**Fig. 1:**
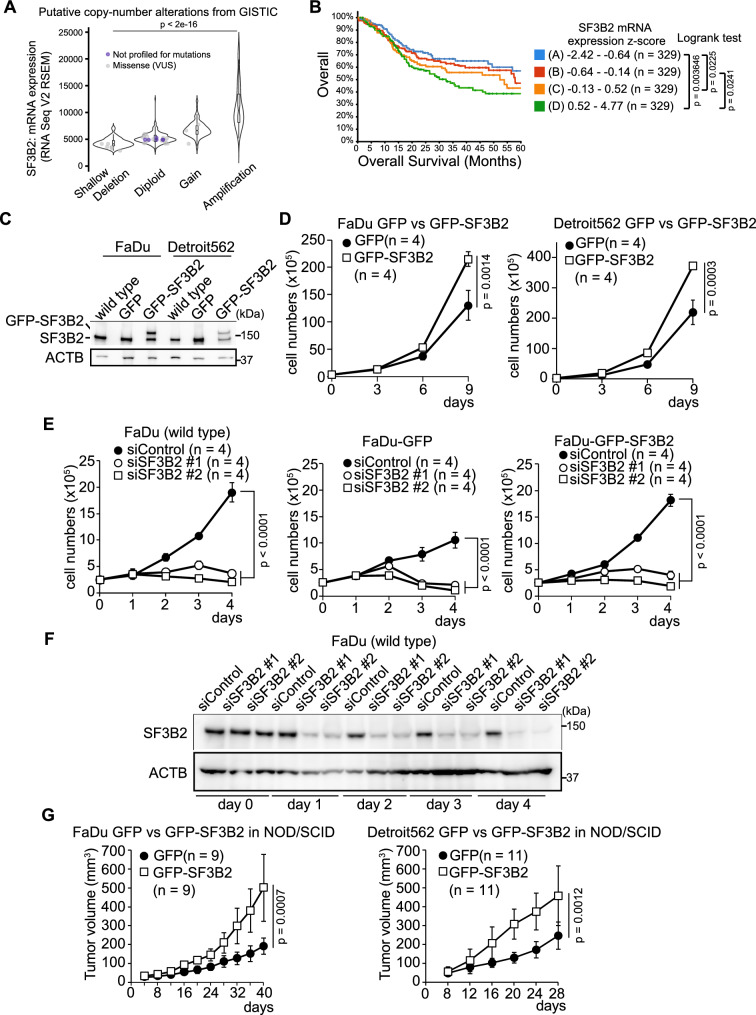
SF3B2 promotes tumor progression in patients with HNSCC and in mouse xenograft HNSCC models. **A** Violin plots of correlation between putative copy-number alterations and SF3B2 mRNA expression. HNSCC data from 7 studies were analyzed using cBioPortal [62]. Grey dot: missense mutation with a variant of uncertain significance (VUS); blue dot: not profiled for mutations. Shallow deletion, n = 136; diploid, n = 738; gain, n = 360; amplification, n = 68. P value was calculated using the pairwise Wilcox rank sum test and adjusted with Bonferroni correction. **B** Kaplan–Meier plot of overall survival in patients with HNSCC. HNSCC data from 7 studies were analyzed using cBioPortal [62]. Patients were stratified into four groups according to SF3B2 expression. **C** Western blots of SF3B2 and ACTB in wild type and GFP or GFP-SF3B2 stably-expressing FaDu and Detroit562 cells. **D** Line plots of cell proliferation of GFP or GFP-SF3B2 stably-expressing FaDu and Detroit562 cells. P value was calculated using the Student’s *t*-test. **E** Line plots of cell proliferation of siControl or two different siSF3B2-treated FaDu, GFP-FaDu, and GFP-SF3B2-FaDu cells. P value was calculated using the Tukey–Kramer test. **F** Western blots of SF3B2 and ACTB in siControl or two different siSF3B2-treated wild-type FaDu cells. **G** Line plots of tumor growth of GFP-FaDu and GFP-SF3B2-FaDu cells or GFP-Detroit562 and GFP-SF3B2-Detroit562 cells in NOD-SCID mice. P value was calculated using Welch’s *t*-test

To determine how SF3B2 contributes to increasing malignancy in solid tumors aside from CRPC, we aimed to elucidate the functions of SF3B2 in HNSCC. We reanalyzed clinical data from previous studies using cBioPortal [16–20]. Our results showed that SF3B2 mRNA expression was positively correlated with the amplification of copy number variations, but not with mutations in SF3B2 (p < 2e−16, Fig. 1A), in patients with HNSCC. Overall survival was negatively correlated with SF3B2 expression level (p = 0.0123, Fig. 1B). On the other hand, SF3B2 expression positively correlated with the neoplasm histologic grade (q = 5.636e−6, Additional file 1: Fig. S1A) but not with HPV status (q = 0.145, Additional file 1: Fig. S1B), alcohol consumption frequency (q = 0.322, Additional file 1: Fig. S1C), or cigarette smoking history (q = 0.354, Additional file 1: Fig. S1D).

Next we established SF3B2 stably-expressing HNSCC cells using FaDu (GFP-FaDu and GFP-SF3B2-FaDu) and Detroit562 (GFP-Detroit562 and GFP-SF3B2-Detroit562) cells [21, 22] (Fig. 1C). High SF3B2 expression increased the proliferation of the two cell lines in vitro (FaDu, p = 0.0014; Detroit562, p = 0.0003, Fig. 1D). In contrast, SF3B2 knockdown using two specific siRNAs (siSF3B2) consistently suppressed cell proliferation in FaDu cells (p < 0.0001, Fig. 1E). The two SF3B2 siRNAs decreased SF3B2 protein expression by the same degree (Fig. 1F). High SF3B2 expression promoted tumor growth of xenograft HNSCC in mouse models (p = 0.0007, FaDu; p = 0.0012, Detroit562, Fig. 1G). These results suggest that high SF3B2 expression is induced by copy number amplification and promotes HNSCC progression.

### SF3B2 interacts with gene regulatory elements and RNA

**Fig. 2:**
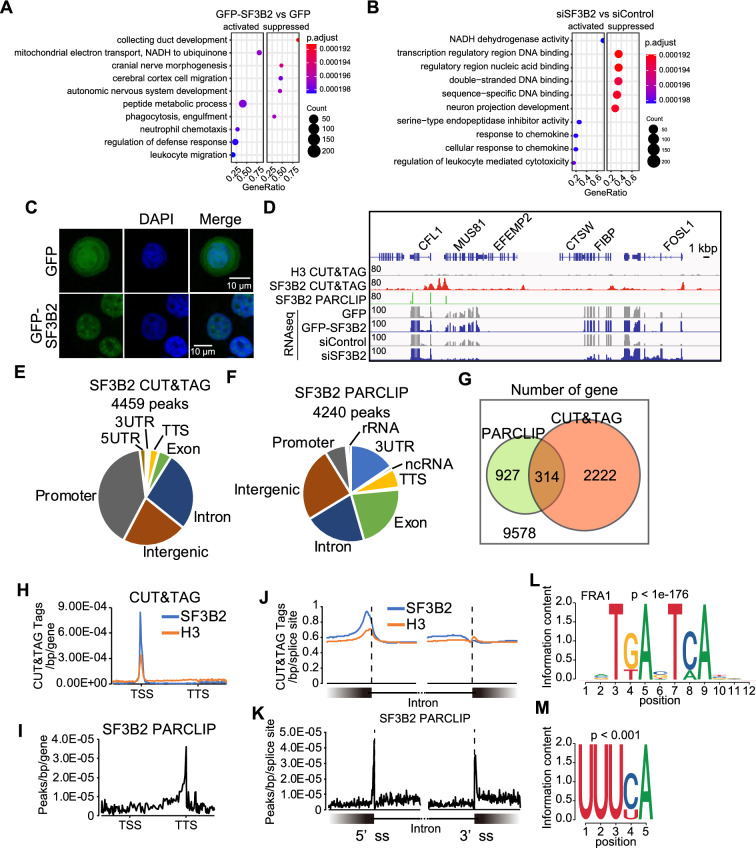
SF3B2 interacts with gene regulatory elements and RNA. **A**, **B** Dot plot of Gene set enrichment analysis between GFP- and GFP-SF3B2-FaDu cells **A** or between siControl and siSF3B2-treated FaDu cells **B**. The top five gene ontologies (GO) enriched in the activated or suppressed genes were presented. **C** Microscopic images of GFP- and GFP-SF3B2-FaDu cells. **D** Sequencing tracks of H3 and SF3B2 CUT&Tag in FaDu cells, SF3B2 PAR-CLIP in SF3B2-tandem affinity purification (TAP) tag stably-expressing FaDu cells, RNA-seq in GFP- and GFP-SF3B2-FaDu cells, and RNA-seq in siControl- and siSF3B2-treated FaDu cells. **E** Pie chart of 4459 peaks of SF3B2-binding genomic regions. **F** Pie chart of 4240 peaks of SF3B2-binding RNA regions. **G** Overlap of gene number between SF3B2-DNA binding genes and SF3B2–RNA binding genes. **H**–**I** Metagene plot of SF3B2 and H3 CUT&Tag peaks **H** or SF3B2 PAR-CLIP peaks **I**. TSS, transcription start site; TTS, transcription termination site. **J**–**K** The intron and exon junction-centered plots of SF3B2 and H3 CUT&Tag signals **J** or SF3B2 PAR-CLIP signals **K**. 5′ss, 5′ splice site. 3′ss, 3′ splice site. **L** Representative motifs in SF3B2 CUT&Tag peaks. The motif was detected in 16.17% of SF3B2 peaks. **M** Representative motifs in SF3B2 PAR-CLIP peaks. The motif was detected in 29% of the SF3B2-binding RNA region

To elucidate how SF3B2 promotes tumor proliferation in HNSCC, gene expression profiles were analyzed using RNA sequencing (RNA-seq) in SF3B2-overexpressing (GFP-SF3B2-FaDu) and SF3B2-depleted (siSF3B2-treated) FaDu cells. Gene Ontology (GO) analysis revealed that SF3B2-overexpressing cells had increased expression of genes related to mitochondrial electron transport (Fig. 2A). Conversely, SF3B2-depleted cells had decreased expression of genes related to transcription regulatory region DNA binding (Fig. 2B), suggesting that high SF3B2 expression promotes tumor cell proliferation by changing the gene expression profile.

Immunofluorescence imaging of GFP-SF3B2-expressing FaDu cells showed that SF3B2 was localized in the nucleus but not in the nucleolus (Fig. 2C), implying that SF3B2 binds to RNA and chromatin. We applied Cleavage Under Targets and Tagmentation (CUT&Tag) [23] and photoactivatable ribonucleoside-enhanced cross-linking immunoprecipitation (PAR-CLIP) analyses to evaluate binding of SF3B2 to chromatin and RNA regions (Fig. 2D and Additional file 1: Fig. S2). PAR-CLIP provides information on the direct interaction between a target protein and RNA [24]. SF3B2 PAR-CLIP showed clear peaks, as previously reported [6]. Notably, SF3B2 CUT&Tag also indicated binding of SF3B2 to chromatin, compared to H3 CUT&Tag signals (Fig. 2D). SF3B2 CUT&Tag identified 4459 peaks that flanked the promoter, intergenic, and intron regions (Fig. 2E). The SF3B2–RNA binding detected by SF3B2 PAR-CLIP showed 4240 peaks, but it differed from the CUT&Tag profile with more peaks detected at the 3’UTR and exons (Fig. 2F). Comparative analysis of the gene expression data from SF3B2–chromatin and SF3B2–RNA binding showed that 2222 genes were solely associated with SF3B2–chromatin binding, 927 genes were solely associated with SF3B2–RNA binding, and 314 genes were associated with SF3B2 binding to both chromatin and RNA (Fig. 2G). Metagene analysis indicated distinct profiles for SF3B2–chromatin and SF3B2–RNA binding; the SF3B2 signal was accumulated around the TSS upon chromatin binding (Fig. 2H) and around transcription termination site (TTS) upon RNA binding (Fig. 2I). Moreover, by aligning reads at 5'splice site (5′ss) and 3′ splice site (3'ss) junction as the center, we observed that the SF3B2 signal was moderately enriched at the edge of the exon at the 5′ss on chromatin (Fig. 2J). In contrast, SF3B2 signal accumulated at the 5′ss and 3′ss on RNA (Fig. 2K). FRA1 is encoded by *FOS-like 1 (FOSL1)* and recognizes the DNA motif; it was enriched at SF3B2-binding genomic regions (16.17% of SF3B2 peaks, p < 1e-176, Fig. 2L). On the other hand, the UUUCA motif was enriched at SF3B2-binding RNA regions (29% of SF3B2 peaks, p < 0.001, Fig. 2M), suggesting that SF3B2 binds differently to DNA and RNA target regions. These data suggest that SF3B2 has a dual function in gene regulatory elements and RNA.

### SF3B2 modulates Pol2 activity

**Fig. 3:**
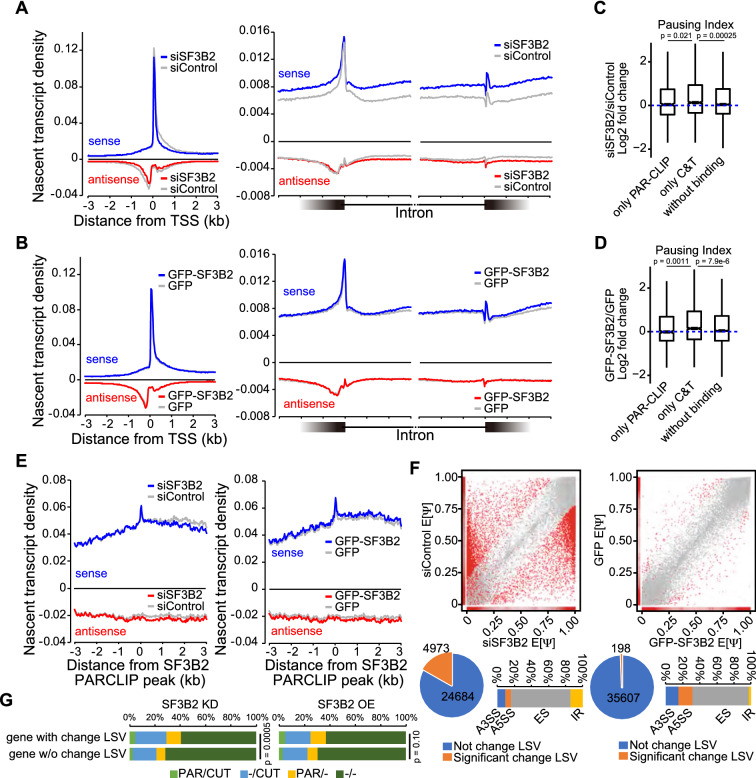
SF3B2 modulates RNA polymerase II activity. **A**, **B** Line plot of nascent transcript density around TSS, 5'ss, and 3'ss in siSF3B2 and siControl-treated FaDu cells **A** or GFP- and GFP-SF3B2-FaDu cells **B**. The nascent transcript was analyzed using PRO-seq. **C**, **D** Pausing index of Pol2 in siSF3B2 and siControl-treated FaDu cells **C** or GFP- and GFP-SF3B2-FaDu cells **D**. Only PAR-CLIP, genes with SF3B2 peaks on RNA but not chromatin, n = 901. Only CT, genes with SF3B2 peaks on chromatin but not RNA, n = 2202. Without binding, genes without SF3B2 peaks, n = 7780. P value was calculated using the pairwise Wilcox rank sum test adjusted with Bonferroni correction. **E** Line plot of nascent transcript density centering SF3B2 PAR-CLIP peaks. **F** Dot plot of percent spliced in the index (Psi/Ψ). The red dot indicates the significantly changed local splicing variation (LSV). Pie chart of LSV with significant and non-significant change. A 100% stacked bar plot of the significantly changed LSV. A3SS, alternative 3′ splice site; A5SS, alternative 5′ splice site; ES, exon skipping; IR, intron retention. **G** A 100% stacked bar plot of genes stratified by SF3B2 PAR-CLIP and CUT&Tag peaks with or without the significantly changed LSV. Genes were stratified into four categories: genes with SF3B2 PAR-CLIP and CUT&Tag peaks (PAR/CUT); genes with CUT&Tag peaks (−/CUT); genes with SF3B2 PAR-CLIP peaks (PAR/−); genes without SF3B2 peaks (−/−). SF3B2 KD, knockdown of SF3B2. SF3B2 OE, GFP-SF3B2 overexpression. P value was calculated using the Fisher’s Exact test with Monte Carlo simulation

Next, we used precision nuclear run-on and sequencing (PRO-seq) to determine whether SF3B2 is involved in transcription regulation [25] (Additional file 1: Fig. S3A). SF3B2 depletion in FaDu cells modestly decreased the nascent transcript density of all genes at TSS (Fig. 3A). In contrast, SF3B2 depletion increased the density of nascent transcripts around the exon–intron junction in the sense strand. SF3B2 depletion modestly increased nascent transcript density in the last exon before TTS (Additional file 1: Fig. S3B). SF3B2 overexpression did not change the nascent transcript profile at the TSS and the exon–intron junction (Fig. 3B) and modestly increased the nascent transcripts after the TTS (Additional file 1: Fig. S3B). We then stratified genes based on SF3B2 binding to chromatin and RNA, to determine whether SF3B2 modulated the promoter-proximal pausing of Pol2. The Pol2 pausing step is critical for transcription [26]. SF3B2 depletion, as well as SF3B2 overexpression, slightly increased the pausing index of genes associated with SF3B2–chromatin binding but not of genes associated with SF3B2–RNA binding (p = 0.021 for siSF3B2, p = 0.0011 for GFP-SF3B2, Fig. 3C, D). In contrast to the function SF3B2 in gene regulation via chromatin binding, SF3B2–RNA binding did not change nascent transcript density near SF3B2–RNA binding sites in SF3B2-depleted or SF3B2-overexpressing cells (Fig. 3E). SF3B2 depletion disrupted the control of 4973 local splicing variations (LSVs), while SF3B2 overexpression had a moderate effect on LSVs (Fig. 3F), as was previously reported [6]. Approximately 50% of the significantly changed LSVs contained exon skipping (ES) (Fig. 3F). SF3B2 depletion was associated with changing more LSVs in genes associated with binding of SF3B2 to chromatin, RNA, or both, compared to genes without SF3B2-binding (p = 0.0005), while high SF3B2 expression did not show any difference (p = 0.10, Fig. 3G). These data suggest that SF3B2 is involved in the regulation of transcription and directly and indirectly controls RNA splicing.

### Correlation of binding of SF3B2 to chromatin and RNA with the level of gene expression

**Fig. 4:**
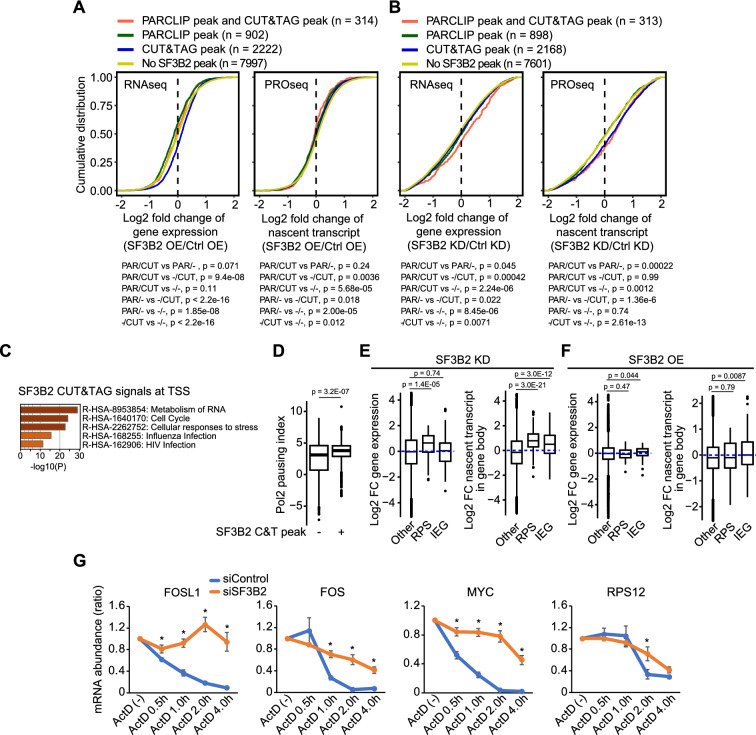
SF3B2-binding to chromatin and RNA are differently correlated with the level of mRNA expression. **A**, **B** Cumulative distribution plots of gene expression changes or nascent transcript expression in GFP-SF3B2-expressing **A** or siSF3B2-treated **B** FaDu cells. Genes were stratified into four categories: genes with SF3B2 PAR-CLIP and CUT&Tag peaks (PAR/CUT); genes with SF3B2 PAR-CLIP peaks (PAR/−); genes with CUT&Tag peaks (−/CUT); genes without SF3B2 peaks (−/−). P value was calculated using the Wilcox rank sum test. **C** Representative GO enrichment of SF3B2 CUT&Tag signals at 909 TSSs with the score of sum of the SF3B2 signal from − 200 to 2000 around TSS and log2 fold change > 2 in comparison to H3. **D** Box plots of Pol2 pausing index in genes with (n = 1077) or without (n = 27,097) SF3B2 CUT&Tag signal at TSS. P value was calculated using the Wilcox rank sum test. **E**, **F** Box plots of log2 fold change of gene and nascent transcript expression upon siSF3B2 treatment **E** or GFP-SF3B2 expression **F**. RPS, Ribosomal protein S genes; IEG, immediate early genes; Other, other genes from RPS and IEG. **E** Left: Other (n = 18,476), RPS (n = 93), and IEG (n = 114). Right: Other (n = 27,818), RPS (n = 157), and IEG (n = 199). **F** Left: Other (n = 17,440), RPS (n = 93), and IEG (n = 114). Right: Other (n = 27,818), RPS (n = 157), and IEG (n = 199). P value was calculated using the Wilcox rank sum test. **G** Line plots of mRNA abundance. Each value was normalized to non-treated as 1.0 and then to 18S RNA. Error bars indicate mean ± SD, n = 3 biologically independent samples. * P < 0.001. P value was calculated using the Student’s *t*-test

We evaluated the effect of SF3B2 binding to chromatin (PRO-seq) and RNA (RNA-seq) on gene and nascent transcript expression levels. SF3B2 overexpression in FaDu cells increased the expression of genes associated with SF3B2–chromatin binding (p < 2.2e−16) and decreased the expression of genes associated with SF3B2–RNA binding (p = 1.85e−08), compared to the expression levels without SF3B2 binding (Fig. 4A). The effect of SF3B2 overexpression on the nascent transcript expression was minimal, but it slightly decreased the expression of the nascent transcripts associated with SF3B2 binding, compared to that of the nascent transcripts not associated with SF3B2 binding (Fig. 4A). Conversely, SF3B2 depletion increased the expression of genes associated with both SF3B2–RNA binding (p = 8.45e−06) and binding of SF3B2 to chromatin and RNA (p = 2.24e−06, Fig. 4B). Depletion of SF3B2 increased the expression of nascent transcripts from genes associated with SF3B2 binding to chromatin and RNA (p = 0.0012) and genes associated with SF3B2–chromatin binding (p = 2.61e−13, Fig. 4B). These results suggest that SF3B2 plays a distinct role in the regulation of gene expression through RNA splicing.

As SF3B2 was involved in the regulation of nascent transcript expression, we investigated the function of SF3B2-binding on chromatin, particularly at TSS, by analyzing 909 select genes associated with binding of SF3B2 to TSS. GO enrichment analysis of these 909 genes revealed that SF3B2 binds to the TSS regions of genes that are involved in the metabolism of RNA and cellular responses to stress (Fig. 4C). SF3B2 binding to TSS increased the Pol2 pausing index (p = 3.2e−07) (Fig. 4D), suggesting that SF3B2 promotes the recruitment of Pol2 to TSS and/or suppresses Pol2 elongation. The ribosomal protein S (RPS) gene is one of the representative genes in the metabolism of RNA, and the immediate early gene (IEG) is involved in cellular responses to stress (Additional file 2: Table S2). SF3B2 depletion increased gene expression of RPS (p = 1.4E-05), but not IEG (p = 0.74), while also increasing the RPS (p = 3.0e−21) and IEG (p = 3.e−12) nascent transcript expression (Fig. 4E). Conversely, SF3B2 overexpression did not significantly affect RPS and IEG expression (Fig. 4F). To further confirm the effect of SF3B2 on RNA, we performed an RNA stability assay using actinomycin D. SF3B2 depletion significantly enhanced RNA stability of *FOSL1*, *FOS*, *MYC*, and *RPS12* (Fig. 4G). These data suggest that SF3B2 promotes Pol2 pausing to modulate RPS transcription and modulates RNA stability.

### SF3B2 promotes binding of SMC1A and CTCF to the SF3B2-binding genomic elements and modulates SMC1A and CTCF transcription activity

**Fig. 5:**
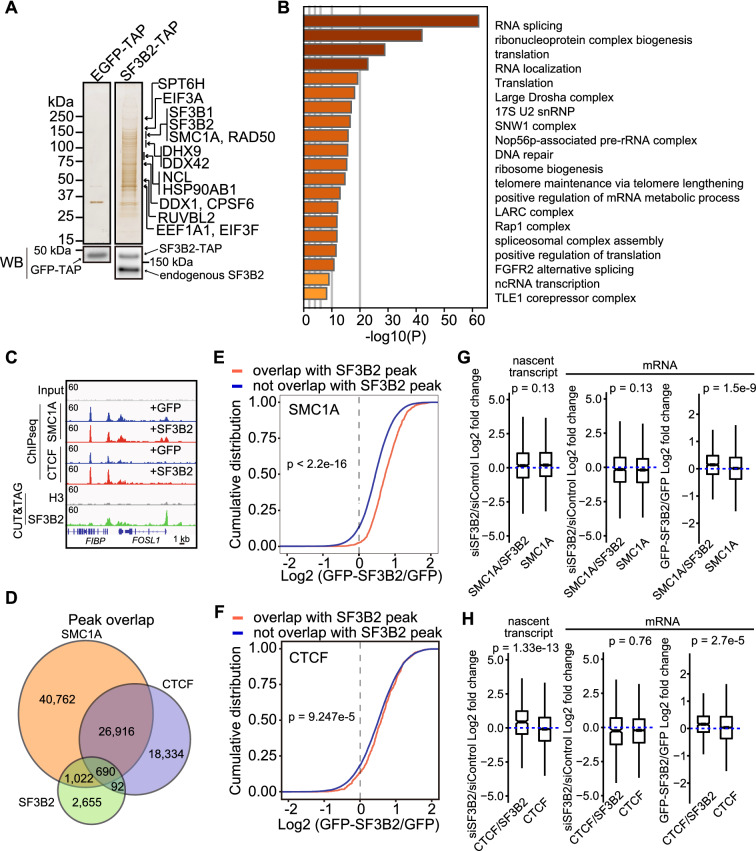
SF3B2 promotes SMC1A and CTCF binding to the SF3B2-binding chromatin regions and modulates SMC1A and CTCF activity. **A** Silver staining of SDS-PAGE gel with SF3B2-TAP complex components. GFP-TAP was used as control. Proteins were identified using MS/MS. Western blotting shows GFP-TAP, SF3B2-TAP, and endogenous SF3B2 protein expression in FaDu cells. The proteins detected in all three independent experiments are shown. **B** SF3B2-associated proteins detected by GO enrichment analysis in at least two out of three independent experiments. GO enrichment was analyzed using Metascape [40]. **C** Sequencing tracks of SMC1A and CTCF ChIP-seq signals and SF3B2 and H3 CUT&Tag signals in GFP- and GFP-SF3B2-FaDu cells. **D** Overlap of peaks among SMC1A, CTCF, and SF3B2. **E** Cumulative distribution plot of SMC1A binding levels (GFP-SF3B2/GFP) at peaks with or without overlap with SF3B2 peaks. SMC1A peaks overlapping with SF3B2 peaks, n = 1389. SMC1A peaks not overlapping with SF3B2 peaks, n = 43,573. **F** Cumulative distribution plot of CTCF binding levels (GFP-SF3B2/GFP) at peaks with or without SF3B2 overlaps. CTCF peaks overlapping with SF3B2 peaks, n = 617. CTCF peaks not overlapping with SF3B2 peaks, n = 24,237. **G** Box plots showing expression of the nascent transcripts and genes with SMC1A and SF3B2 peaks, or with only SMC1A peaks in SF3B2-depleted or SF3B2-overexpressing FaDu cells. Data expressed as fold change in expression compared to respective controls. Genes with SMC1A and SF3B2 peaks, n = 948. Genes with only SMC1A peaks, n = 7787. **H** Box plots showing expression of the nascent transcripts and genes with CTCF and SF3B2 peaks, or with only CTCF peaks in SF3B2-depleted or SF3B2-overexpressing FaDu cells. Data is expressed as fold change in expression compared to respective controls. Genes with CTCF and SF3B2 peaks, n = 423. Genes with only CTCF peaks, n = 6045

To further understand how SF3B2 modulates transcription, SF3B2-associated proteins were purified, using a tandem affinity purification (TAP) tag, from FaDu cells stably expressing SF3B2-TAP and EGFP-TAP (control); subsequently, the proteins were analyzed by mass spectrometry (Fig. 5A). SF3B2 protein was associated with several proteins related to RNA splicing and translation (Fig. 5B and Additional file 1: Fig. S4), which is consistent with the fact that SF3B2 is a component of the SF3b complex [6, 27, 28]. SMC1A, a cohesin component, was detected in all three independent experiments of SF3B2-TAP complex purification (Fig. 5A). Cohesin is a critical factor in chromatin architecture and gene expression [13]. CTCF is a well-known chromatin architectural factor that forms a complex with cohesin [29]. Hence, we hypothesized that SF3B2 is involved in binding of cohesin and CTCF to chromatin and their transcription activity. We compared the sequencing tracks from ChIP-seq analysis for SMC1A and CTCF with the tracks from CUT&Tag analysis for SF3B2 and H3, derived from GFP- (control) and GFP-SF3B2- expressing FaDu cells. The results showed that the SMC1A- and CTCF-binding regions considerably overlapped with the SF3B2-binding regions (Fig. 5C). Approximately 40% of SF3B2-binding regions overlapped with SMC1A-binding regions and approximately 20% overlapped with CTCF-binding regions (Fig. 5D). We detected the enrichment of the FRA1-recognizing motif in SF3B2 and SMC1A overlapping peaks in GFP-FaDu cells (rank 2; p = 1e−82; 1692 target sequences; Additional file 1: Fig. S4B) and in SF3B2 and SMC1A overlapping peaks where SF3B2 overexpression increased counts of SMC1A 1.5-fold or more (rank 1; p = 1e−45 in GFP-SF3B2 FaDu cells; 886 target sequences; Additional file 1: Fig. S4C). We also detected the enrichment of the CTCF motif in SF3B2 and SMC1A overlapping peaks in GFP-FaDu cells (rank 1; p = 1e−108; 1692 target sequences; Additional file 1: Fig. S4D). Moreover, high SF3B2 expression increased SMC1A-binding levels in the SF3B2-binding chromatin regions (p < 2.2e−16, Fig. 5E), whereas the CTCF-binding levels were not significantly affected (p < 9.247e−5, Fig. 5F) without an increase of SMC1A protein expression by high SF3B2 expression (Additional file 1: Fig. S4E). These results suggest that SF3B2 plays a role in recruiting cohesin to the SF3B2-binding chromatin regions.

Finally, we examined gene and nascent transcript expression levels to elucidate whether SF3B2 modulates SMC1A and CTCF transcriptional activity. SF3B2 depletion did not change the nascent transcript and mRNA expression of genes associated with SMC1A- and SF3B2-binding or genes solely associated with SMC1A-binding (p = 0.13, both); in contrast, high SF3B2 expression increased the expression of genes associated with both SMC1A- and SF3B2-binding, compared to the expression level associated with only SMC1A-binding (Fig. 5G). On the other hand, SF3B2 depletion increased the nascent transcript expression of genes associated with both CTCF- and SF3B2-binding, compared to those associated with only CTCF-binding (p = 1.33e−13) but the gene (mRNA) expression was unchanged (p = 0.76). High SF3B2 expression increased the expression of genes associated with both CTCF- and SF3B2-binding, compared to those associated solely with CTCF-binding (p = 2.7e−5, Fig. 5H). These data suggest that SF3B2 negatively modulates CTCF nascent transcription activity and positively modulates gene expression associated with cohesin and CTCF.

## Discussion

Collectively, the findings of this study revealed that SF3B2, an SF3b component involved in the U2 spliceosome, modulates transcription by recruiting SMC1A and regulating SMC1A and CTCF activities and RNA stability. This molecular mechanism could be involved in malignant progression in patients with HNSCC expressing high levels of SF3B2.

We have previously demonstrated that SF3B2 is critical for increasing AR-V7 splicing and promoting malignancy in CRPC [6]. High SF3B2 expression modulates the gene expression profile in CRPC, where AR-V7 is the central transcription factor regulating gene expression. However, AR-V7 is specifically expressed in prostate cancer, whereas high SF3B2 expression is associated with a poor prognosis in patients with at least six types of cancer. High SF3B2 expression also causes minor changes in RNA splicing in genes other than AR-V7 in prostate cancer, suggesting other roles for SF3B2 in gene expression. In the present study, we found that SF3B2 is involved in regulating transcription, as well as the transcription activity of cohesin and CTCF. Compared to the SF3B2 complex in the CRPC cell line [6], SF3B2 in HNSCC cell line was distinctly associated with several chromatin regulating factors belonging to the BAF (SWI/SNF) complex (Fig. 5 and Additional file 1: Fig. S4). The FRA1-recognizing motif was enriched in SF3B2-binding chromatin. FRA1 is a transcription factor encoded by *FOSL1*. High FRA1 expression is associated with poor prognosis in patients with oral squamous cell carcinoma (OSCC), a subtype of HNSCC [30]. This implies that SF3B2 is involved in FRA1-mediated transcriptional regulation. Consistent with SF3B2-binding to chromatin, SRSF2 has recently been reported to form liquid-like condensates with Pol2, and it binds to chromatin at the gene body and 3’ downstream [31]. A different RNA motif was enriched in SF3B2-binding RNA regions in the HNSCC cell line, compared to the SF3B2-recognizing RNA motif in CRPC. This suggests that SF3B2 may form complexes with factors other than RNA splicing factors in a cell type-dependent manner, as SF3B2 has a disordered structure and may change the protein structure, depending on the associated proteins.

Chromatin modifications, such as H3K36me3 and DNA methylation, and CTCF modulate RNA splicing by changing the transcription activity and recruiting an RNA splicing factor [15, 32]. Binding of CTCF to an exon decreases transcription activity and promotes exon inclusion. In this study, knockdown of SF3B2 increased the transcription activity around the exon–intron and intron–exon junctions, suggesting that SF3B2 suppressed the transcription activity around the junctions to execute precise RNA splicing (Fig. 3). Knockdown of SF3B2 increased exon skipping. These findings indicate that SF3B2 is involved in transcription regulation. Although the molecular mechanisms are not entirely clear, we found that SF3B2 increases the recruitment of cohesin and CTCF to the SF3B2-binding genomic regions (Fig. 5). Cohesin and CTCF are crucial factors in organization of chromatin architecture and gene expression [33]. SF3B2 may modulate gene expression levels by tuning the transcription activity of cohesin and CTCF.

Our results showed that SF3B2 interacts with both chromatin and RNA. Yin Yang 1 (YY1) interacts with regulatory chromatin elements and RNA transcribed from the elements to enhance the anchoring of YY1 [34]. However, the SF3B2-binding profiles were different from those of YY1. SF3B2 bound to chromatin at the promoter element, while SF3B2 binding to RNA was often detected near TTS (Fig. 2). SF3B2 binding to chromatin contributed to the activation of gene expression, while SF3B2 binding to RNA contributed to a decrease in gene expression (Fig. 4). SF3B2 was also involved in the regulation of RNA stability. This suggests an SF3B2-mediated feedback loop between transcription activity and mRNA levels. We previously reported that a transcription factor constructs a promoter-TTS loop to regulate transcription termination [35]. Collectively, it can be deduced that a SF3B2-mediated communication loop between the promoter and TTS may regulate the level of mRNA expression.

Our study has some limitations. We could not approach the structural analysis of SF3B2 in cell-type-dependent SF3B2 complexes. SF3B2 is a disordered protein; thus, it is challenging to determine the structure of SF3B2 [27]. Recently, the molecular structure of 17S U2 snRNP with the SF3b complex has been determined [5]. The study revealed that SF3B1 and TAT-SF1 might regulate the disordered domain of SF3B2 in 17S U2 snRNP. This suggests a possibility for determining the position of SF3B2 and SF3B2 structural information by isolating the SF3B2 complex from HNSCC cells. However, doing so would be challenging because SF3B2 might participate in several steps of transcription involving chromatin and RNA. As SF3B2 is a ubiquitously expressed gene, it is unclear whether SF3B2 forms various complexes depending on the cell-type context. It is also unknown whether other SF3b components, such as SF3B1, have a role in chromatin transcription. Thus, further studies are required to fully understand how SF3B2 modulates both transcription and RNA splicing to promote tumor progression in patients with HNSCC.

**Fig. 6:**
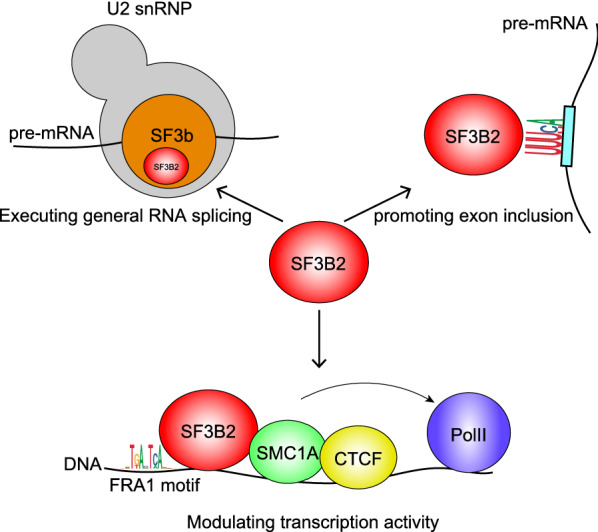
Proposed model of SF3B2 functions in RNA splicing and transcription. The model shows the three SF3B2 functions: (1) executing general RNA splicing as a component of U2 snRNP, (2) promoting exon inclusion through direct binding to RNA, and (3) modulating transcription activity of cohesin and CTCF

In conclusion, results of the previous study and present study clearly indicate that SF3B2 has at least three functions during gene expression (Fig. 6): (1) SF3B2 is involved in the functioning of 17S U2 snRNP as a component of the SF3b complex to execute general RNA splicing. (2) SF3B2 promotes exon inclusion by binding to the pre-mRNA encoding the target exon. (3) SF3B2 binds to genomic regulatory elements that the modulate transcription activity of cohesin and CTCF. In addition, it can be deduced that SF3B2 mediates a feedback loop that may regulate mRNA production through bidirectional transcription and RNA splicing control.

## Materials and methods

### Cell lines and cell culture

Human HNSCC cell lines FaDu and Detroit562 were purchased from ATCC. All cells were confirmed to be *Mycoplasma-*negative before this study (6601, TaKaRa). Cells were cultured in Dulbecco’s modified Eagle’s medium (DMEM, 08,458-45, Nacalai Tesque), supplemented with 10% fetal bovine serum ( FBS, 172012, Sigma), 1% non-essential amino acids (NEAA, 06344-56, Nacalai Tesque), 1% l-glutamine (16948-04, Nacalai Tesque), and 1% penicillin/streptomycin solution (26253-84, Nacalai Tesque), at 37 °C in an atmosphere of 95% relative humidity and 5% CO_2_.

### Transfection

FaDu and Detroit562 cells were transfected with pCAGIP-GFP, pCAGIP-GFP-SF3B2, pCAGIP-GFP-TAP, or pCAGIP-SF3B2-TAP [6] using Fugene HD transfection reagent (E2311, Promega). Cells were seeded into six-well plates (2 × 10^5^ cells/well), to which 3 µg/well DNA plasmid in Opti-MEM (31985070, Gibco) and 9 µL/well Fugene HD were added. To establish GFP- or GFP-SF3B2 stably-expressing FaDu and Detroit562 cells, 3000 transfected cells were cultured in medium containing puromycin (0.2 µg/mL), in a 10-cm dish, until colonies were formed. Colonies were picked and protein expression was analyzed using western blotting.

### Immunofluorescence imaging

GFP- or GFP-SF3B2-expressing FaDu cells (3 × 10^6^) were seeded on cover glass in six-well plates. After 48 h, the cells were washed with PBS and fixed with 4% paraformaldehyde (PFA, 26126-25, Nacalai Tesque) for 5 min at room temperature. After three washes with PBS, the cells were stained with DAPI (1:10000) (10236276001, Roche) for 5 min at room temperature. Next, they were washed with ultrapure water and mounted in Vector Shield (H-1000, Vector Laboratories). Immunofluorescence staining images were obtained using a confocal laser microscope FV1200 (OLYMPUS).

### Western blotting

Proteins were extracted from 1×10^5^ cells in 10 µL of sample buffer (1610737, BIO-RAD). The extracted proteins were separated in a 5%–20% polyacrylamide gradient gel (191-15011, Wako) and transferred onto a polyvinylidene difluoride membrane. The membrane was blocked with 3% skim milk at room temperature for 1 h and then incubated with anti-SF3B2 (sc-514976, Santa Cruz, 1:200 dilution) or anti-beta actin (A5441, Sigma, 1:5000 dilution) antibodies. Then, after washing twice and blocking with 3% skim milk, the membrane was incubated with an HRP-conjugated mouse secondary antibody. The signals were detected with Chemi-Lumi One or Chemi-Lumi One Super (02230-30, Nacalai Tesque) using an ImageQuant LAS 4000 mini system (GE Healthcare).

### siRNA treatment

The following pre-designed MISSION siRNAs were purchased from Sigma-Aldrich: siSF3B2 #1, Hs_SF3B2_6217_s (5-GUAUGUGACUGAAGAACCU-3) and Hs_SF3B2_6217_as (5-AGGUUCUUCAGUCACAUAC-3); siSF3B2 #2, Hs_SF3B2_6219_s (5-GAUUGAGUAUGUGACUGAA-3) and Hs_SF3B2_6219_as (5-UUCAGUCACAUACUCAAUC-3); and siControl (SIC-001, SIGMA). siSF3B2 #2 (Hs_SF3B2_6219) was used for RNA-seq and PRO-seq. A mixture of 30 pmol siRNA in 500 µL Opti-MEM (31985070, Gibco) and 5 µL Lipofectamine RNAiMAX Transfection Reagent (13778150, Thermo Fisher Scientific) was added into each well, and then FaDu cells were seeded (2.5 × 10^5^ cells/well) into six-well plates. The transfected cells were incubated at 37 °C in an atmosphere of 95% relative humidity and 5% CO_2_ for 48 h.

### Human HNSCC xenograft model

The Osaka University Animal Experiments Committee approved all experiments using mice, and all experiments were performed in accordance with their guidelines. The in vivo tumor growth of human HNSCC cells was examined using a subcutaneous xenograft model. Cancer cells (2 × 10^6^ cells) in 50 µL PBS were transplanted into the flanks of 8-week-old NOD/SCID mice (Charles River) under deep anesthesia. The mice were maintained and handled according to approved protocols and the guidelines of the Animal Committee of Osaka University (Osaka, Japan), as previously described [6]. Tumor size was measured once every four days and the tumor volume was calculated according to the following formula: tumor volume (mm^3^) = length × (width)^2^/2.

### RNA-seq

Sequencing libraries from at least two biological replicate RNA samples were prepared using the NEBNext Ultra RNA Library Prep Kits for Illumina (E7530L, NEB) following the manufacturer's instructions, as previously described [6]. mRNA was enriched using NEBNext Oligo d(T)25 beads. The sequencing libraries were analyzed by HiSeq X Ten (Illumina).

#### PAR-CLIP

PAR-CLIP was performed using a previously published protocol [24], with some modifications [6, 36]. Briefly, FaDu cells stably expressing SF3B2-TAP were labeled with 100 µM 4-SU (T4509, Sigma) and then cross-linked by irradiation with 365 nm UV at 150 mJ/cm^2^. The whole-cell lysate was collected and treated with 5 U/mL RNase T1 (EN0541, Fermentas) for 15 min at 22 °C, as described previously [37]. TAP tag fusion proteins were immunoprecipitated via overnight incubation (at 4 °C) with IgG Sepharose 6 Fast Flow (17-0969-01, GE Healthcare). The beads were treated with 0.2 U/µL MNase (M0247S, NEB) at 37 °C for 5 min and then with 0.5 U/µL calf intestinal alkaline phosphatase (M0525S, NEB) at 37 °C for 10 min. Subsequently, a 3′ linker was ligated to the RNA fragments by incubating the beads with 0.5 U/µL T4 RNA ligase (EL0021, Thermo Fisher Scientific) overnight at 16 °C, as described previously [38]. The fragmented RNA was radiolabeled using γ-[^32^P]ATP. Proteins covalently bound to radiolabeled RNA were collected and then separated using NuPAGE Novex 4–12% Bis-Tris gels (NP0335BOX, Thermo Fisher Scientific). After the band corresponding to SF3B2-TAP was excised, the radiolabeled RNA was isolated from the RNA–protein complex using proteinase K. A 5′ linker was ligated to the isolated RNA fragments. The linker-ligated RNAs were separated using a 10% TBE-urea gel (EC68752BOX, Thermo Fisher Scientific), and the bands between 70 and 130 nt in length were excised. The extracted RNAs were subjected to RT-PCR, and the resulting PCR products were separated using a 10% TBE gel (Thermo Fisher Scientific). PCR products between 140 and 190 bp in length were excised and eluted. The libraries were sequenced using the Illumina HiSeq X ten platform.

### Tandem affinity purification and mass spectrometry

The SF3B2-TAP-expressing HNSCC cell line was established using FaDu cells. The SF3B2-TAP complex was purified from nuclear extracts using TAP technology, as previously described [39]. The purified proteins were concentrated using Amicon Ultra-0.5 mL 3 K (UFC500308, Merck) and separated via SDS-PAGE. After staining the gel with silver, the protein bands were excised, digested with trypsin in-gel, and analyzed via LC/MS–MS. GO enrichment in the SF3B2-associated proteins, found in two out of three independent experiments, was analyzed using Metascape [40].

### ChIP-seq

ChIP was performed as previously described [41] using anti-CTCF (07-729, Millipore, 10 µL, 1:10 dilution) and anti-SMC1 (A300-055A, BETHYL, 10 µL, 1:10 dilution) antibodies. Sequencing libraries were generated using the NEBNext Ultra II DNA Library Prep Kit (E7103, NEB). The libraries were sequenced using the Illumina HiSeq X ten platform.

### CUT&Tag

CUT&Tag was performed with CUT&Tag-IT Assay Kit (53160, ACTIVE MOTIF) in 1.5 × 10^6^ FaDu cells using anti-SF3B2 (sc-514976, Santa Cruz, 5 µL, 1:20 dilution) and anti-H3 (ab1791, Abcam, 1 µL, 1:100 dilution) antibodies. Rabbit anti-mouse IgG (ab6709, Abcam, 1 µL) was used to enhance the signal. The cells were collected using a cell scraper.

### PRO-seq

PRO-seq was performed as previously described [42]. Briefly, 3 × 10^6^ FaDu cells were collected and washed with ice-cold PBS. *Drosophila melanogaster* S2 cells (10% of the human cell number) were added to each sample as a spike-in control for normalization. The combined cells were resuspended in cold permeabilization buffer [10 mM Tris–HCl, pH 7.4, 300 mM sucrose, 10 mM KCl, 5 mM MgCl2, 1 mM EGTA, 0.05% Tween-20, 0.1% NP40 substitute, 0.5 mM DTT, 1:100 protease inhibitor cocktail, and 4 U/mL SUPERaseIN (Invitrogen)] and incubated on ice. The permeabilized cells were then pelleted, washed twice with permeabilization buffer, and resuspended in ice-cold storage buffer (10 mM Tris–HCl, pH 8.0, 25% glycerol, 5 mM MgCl2, 0.1 mM EDTA, and 5 mM DTT) at a concentration of 2 × 10e7 nuclei per 100 μL. Nuclear run-on (NRO) assays were performed using biotin-11-NTPs. In total, 2 × 10e7 nuclei per 100 μL were thoroughly mixed with an equal amount of pre-heated 2 × NRO reaction mixture [10 mM Tris–HCl, pH 8.0, 5 mM MgCl2, 300 mM KCl, 1 mM DTT, 1% Sarkosyl, 50 μM each of Biotin-11-A/G/C/UTP (PerkinElmer, Waltham, MA), and 0.8 U/μL RNase inhibitor] and incubated at 37 °C for 3 min in a heat block. Nascent RNA was extracted, purified, and fragmented by base hydrolysis in 0.2 N NaOH on ice for 10 min. After neutralization, fragmented nascent RNA was bound to DynabeadsTM M-280 Streptavidin magnetic beads (Invitrogen) and incubated for 20 min at 4 °C. The beads were sequentially washed as follows: twice in high-salt buffer (2 M NaCl, 50 mM Tris–HCl, pH 7.4, 0.5% Triton X-100), twice in medium salt buffer (300 mM NaCl, 10 mM Tris–HCl, pH 7.4, 0.1% Triton X-100), and once in low-salt buffer (5 mM Tris–HCl, pH 7.4, 0.1% Triton X-100). Biotinylated RNA was extracted from the beads and precipitated using ethanol. The 3′ RNA adaptors were ligated to biotinylated RNA, and the second round of biotin-streptavidin purification was performed. The mRNA cap was then removed, and the reverse 5′ RNA adaptor was ligated. After the third round of biotin-streptavidin purification, adaptor-ligated nascent RNA was reverse-transcribed into complementary DNA (cDNA) using the RP1 primer. cDNA was amplified with index primers, and amplicons of 120–350 bp were selected using AMPure XP beads (Beckman Coulter, Brea, CA). Equimolar concentrations of library fractions were pooled and sequenced using a high-output flow cell on the NovaSeq 6000 platform (Illumina).

### RNA stability assay

Three days after 10 nM siRNA transfection to FaDu cells using Lipofectamine RNAi MAX (13778150, ThermoFisher), the cells were treated with 5 µg/ml actinomycin D for 0.5, 1, 2, and 4 h. RNA was collected using Sepasol-RNA I Super G (09379-55, Nacalai Tesque). The amounts of target RNA were measured using iTaq Universal SYBR Green One-Step Kit (1725151, BioRad) with specific primers as shown below. qRT-PCR was performed using CFX Connect Real-Time PCR Detection System (BioRad). FOSL1 F: GGCCTTGTGAACAGATCAGC and R: AGTTTGTCAGTCTCCGCCTG; MYC F: ACAGCTACGGAACTCTTGTGCGTA and R: CAGCCAAGGTTGTGAGGTTGCATT; FOS F: AGATTGCCAACCTGCTGAAGGAGA and R: TCAGATCAAGGGAAGCCACAGACA; RPS12 F: TCCGTCCTACCGGAAACCTA and R: TTCCAAACAGCAACCCACAC; 18S rRNA F: TCAACTTTCGATGGTAGTCGCCGT and R: TCCTTGGATGTGGTAGCCGTTTCT.

### Statistical analysis

Student’s two-tailed *t*-test was used to compare two parametric samples, and the Tukey–Kramer test was used for comparisons between multiple parametric samples. Welch’s *t*-test was used to compare two non-homoscedastic samples with a normal distribution. Pairwise Wilcox rank sum test adjusted with Bonferroni correction was used for comparing multiple samples. Fisher’s exact test with Monte Carlo simulation was used for Fig. 3G.

### Bioinformatics

#### Tools

awk v4.1.3 (https://www.gnu.org).

Bedtools 2.26.0/2.29.2 [43]

Bowtie 2–2.2.3/2.3.5.1 [44]

cERMIT 1.1 [45]

Cutadapt 1.9.1 [46]

DESeq2 1.26.0 [47]

FASTX-Toolkit 0.0.13 (http://hannonlab.cshl.edu/fastx_toolkit/).

Fastqc 0.11.5 (https://www.bioinformatics.babraham.ac.uk/projects/fastqc/).

HOMER 4.9.1 [48]

IGV 2.9.2 [49, 50]

MAJIQ 0.9.2a [51]

PARalyzer 1.5 [52]

Perl 5.14.2 (https://www.perl.org).

R 3.6.3 (https://www.r-project.org).

Rstudio 1.4.1103 (https://www.rstudio.com).

Samtools 0.1.17/1.7 [53]

STAR 2.5.3a [54]

Stringtie v1.3.4b [55]

TrimGalore 0.6.6/0.64_dev (https://github.com/FelixKrueger/TrimGalore).

ucsc-bigWigAverageOverbed v2 (https://anaconda.org/bioconda/ucsc-bigwigaverageoverbed).

ucsc-wigToBigWig v4 (https://anaconda.org/bioconda/ucsc-wigtobigwig).

ucsc-bedGraphToBigWig v4 (https://anaconda.org/bioconda/ucsc-bedgraphtobigwig).

#### R package

AnnotationDbi v1.48.0 (https://bioconductor.org/packages/release/bioc/html/AnnotationDbi.html).

clusterProfiler v3.14.5 [56]

DOSE v3.12.0 [57]

dplyr v1.0.4 (https://cran.r-project.org/web/packages/dplyr/index.html).

enrichplot v1.6.1 (https://yulab-smu.top/biomedical-knowledge-mining-book/).

ggplot2 v3.3.3 [58] (http://ggplot2.org).

org.Hs.eg.db v3.10.0 (https://bioconductor.org/packages/release/data/annotation/html/org.Hs.eg.db.html).

plyr v1.8.6 [59]

reshape2 v1.4.4 [60]

rtracklayer (version 1.48.0) [61]

tidyr v1.1.2 (https://cran.r-project.org/web/packages/tidyr/index.html).

#### Clinical data analysis

SF3B2 mRNA expression, overall survival, and clinical data analyses in patients with HNSCC were analyzed using cBioPortal [62]. We excluded overlapping samples and patients. 1316 Patients were stratified by SF3B2 mRNA expression (RNA Seq V2 RSEM) level.

#### RNA-seq data analysis

Paired-end reads were mapped using STAR on the human genome reference hg19 after checking the quality using FastQC. Strigtie and DESeq2 were used to merge the results and calculate normalized gene expression. Statistical analysis of gene expression was performed using DESeq2. Sequencing tracks were generated using IGVtools with option -z 7, as previously described [6]. The correlations between replicate samples were analyzed using the cor.test program in R. The local splicing variations were calculated using MAJIQ and plotted using the R ggplot2 package. The cumulative distribution of log2 fold change of expression was calculated using the R plyr package and plotted using the R ggplot2 package. Gene set enrichment analysis was calculated and plotted based on the log2 fold change calculated using DESeq2, using R packages ClusterProfiler, EnrichPlot, org.Hs.eg.db, and DOSE.

#### PRO-seq data analysis

Single-end reads were mapped onto the human reference genome hg19 using bowtie2 after removing the adaptor sequences with the option—end-to-end—no-ununals. Correlations between replicate samples were calculated using the cor.test program in R. The mapped reads were normalized using HOMER. The sequencing tracks were generated using HOMER and IGVtools with the option -z 7. FPKM was calculated using the analyzeRepeats program in HOMER or R package rtracklayer. The TSS-, TTS-, 5′ss-, and 3′ss-centered profiles were calculated using the annotatePeaks program with the option -size 6000 -hist 25 -pc 3. The trimmed reads were mapped to the human genome hg19 and *D. melanogaster* genome dm6 using Bowtie2 to normalize the PRO-seq data based on the *D. melanogaster* spike-in. Read counts were normalized according to the genomic coverage of mapped *Drosophila* reads using bedtools and samtools. The pausing index was calculated using the analyzeRepeats program in HOMER with the option -pc 3 or R package rtracklayer.

#### CUT&Tag and ChIP-seq data analysis

Paired-end reads were mapped to the human reference genome hg19 using bowtie2 with the option—local—sensitive-local—minins 0—maxins 500—no-discordant—no-mixed—fr—no-unal, after checking the quality using trim-galore. The mapped reads were normalized using HOMER. To calculate the correlation between replicate libraries, bigwig files were generated using bedGraphToBigWig. The counts in each bin with 10,000 windows were calculated using bigWigAverageOverBed, and the correlation was calculated using the cor.test program in R with the Pearson method. The sequencing tracks were generated using HOMER and IGVtools with the option -z 7. The SF3B2 CUT&Tag peaks were called using the findPeaks program in HOMER with the option -style factor, compared to H3 CUT&Tag reads. The H3 CUT&Tag, CTCF, and SMC1A peaks were compared to the sonicated genome of FaDu cells. The obtained peaks were annotated using the annotatePeaks program in HOMER. The overlap of peaks was calculated using the mergePeaks program in HOMER. Metagene- and splice junction-centered profiles were calculated using the makeMetaGeneProfile program in HOMER with options rna, splice3p, and splice5p. The motif enriched around SF3B2-binding chromatin regions was determined using the findMotifsGenome program in HOMER with the option -size 450 and rendered using R packages ggplot2 and ggseqlogo.

#### PAR-CLIP data analysis

After removing the adaptor sequences, PAR-CLIP data were analyzed using bowtie2, PARalyzer, and cERMIT, as previously described [6]. The correlation between replicate libraries was calculated as described in the CUT&Tag and ChIP-seq data analysis section. The obtained peaks were annotated using HOMER. Normalized sequencing tracks were generated using the IGV and IGVtools. Metagene- and splice junction-centered profiles were calculated using the makeMetaGeneProfile program. The SF3B2 PAR-CLIP motif was rendered using the R packages ggplot2 and ggseqlogo.

## Supplementary Information

**Additional file 1: Figure S1**. Correlation between SF3B2 expression and clinical information. (**A**-**C**) Cumulative bar chart of neoplasm histologic grade (**A**), HPV status (**B**), and alcohol consumption frequency (**C**). (**D**) Dot plot of person cigarette smoking history pack-year value. Clinical data were analyzed using cBioPortal. **Figure S2**. Autoradiograph of SF3B2 PAR-CLIP. ^32^P-labeled RNA crosslinking-SF3B2-TAP was detected by autoradiograph. Proteins were separated in SDS-PAGE and transferred to the membrane. **Figure S3**. RNA polymerase II activity in SF3B2 knockdown or overexpression. (**A**) Dot plot of correlation between replicate 1 (rep 1) and replicate 2 (rep 2) in each PRO-seq library. (**B**) Line plot of nascent transcript density around TTS in siSF3B2 and siControl-treated FaDu cells or GFP- and GFP-SF3B2-FaDu cells. **Figure S4**. Protein interaction network between SF3B2-associated proteins and DNA-binding motifs. (**A**) Representative protein interaction networks are shown with p value. The network of protein interaction was analyzed using Metascape. (**B**) FRA1 motif in SF3B2 and SMC1A overlapping peaks in GFP-FaDu cells. (**C**) FRA1 motif in SF3B2 and SMC1A overlapping peaks where SF3B2 overexpression increased counts of SMC1A 1.5-fold or more. (**D**) CTCF motif in SF3B2 and SMC1A overlapping peaks in GFP-FaDu cells. (**E**) Western blots of SMC1A and H3 in wild type and GFP or GFP-SF3B2 stably-expressing FaDu cells. **Figure S5**. Original images of western blots and silver staining.

**Additional file 2: **Lists of genes assigned to the RPS or STRESS gene ontology.

## Data Availability

Sequencing data were deposited to DRA012449.
