# Supplementary material for: Dual function of SF3B2 on chromatin and RNA to regulate transcription in head and neck squamous cell carcinoma
Source: Cell Biosci. 2022 Jun 17;12:92. doi: 10.1186/s13578-022-00812-8 (PMC9206271; doi:10.1186/s13578-022-00812-8)
Supplement: Supplementary file 1 — Additional file 1: Figure S1. Correlation between SF3B2 expression and clinical information. (A-C) Cumulative bar chart of neoplasm histologic grade (A), HPV status (B), and alcohol consumption frequency (C). (D) Dot plot of person cigarette smoking history pack-year value. Clinical data were analyzed using cBioPortal. Figure S2. Autoradiograph of SF3B2 PAR-CLIP. 32P-labeled RNA crosslinking-SF3B2-TAP was detected by autoradiograph. Proteins were separated in SDS-PAGE and transferred to the membrane. Figure S3. RNA polymerase II activity in SF3B2 knockdown or overexpression. (A) Dot plot of correlation between replicate 1 (rep 1) and replicate 2 (rep 2) in each PRO-seq library. (B) Line plot of nascent transcript density around TTS in siSF3B2 and siControl-treated FaDu cells or GFP- and GFP-SF3B2-FaDu cells. Figure S4. Protein interaction network between SF3B2-associated proteins and DNA-binding motifs. (A) Representative protein interaction networks are shown with p value. The network of protein interaction was analyzed using Metascape. (B) FRA1 motif in SF3B2 and SMC1A overlapping peaks in GFP-FaDu cells. (C) FRA1 motif in SF3B2 and SMC1A overlapping peaks where SF3B2 overexpression increased counts of SMC1A 1.5-fold or more. (D) CTCF motif in SF3B2 and SMC1A overlapping peaks in GFP-FaDu cells. (E) Western blots of SMC1A and H3 in wild type and GFP or GFP-SF3B2 stably-expressing FaDu cells. Figure S5. Original images of western blots and silver staining. [file 13578_2022_812_MOESM1_ESM.pdf]

SUPPLEMENTARY DATA

Dual function of SF3B2 on chromatin and RNA to regulate transcription in head and neck squamous cell carcinoma

Koji Kitamura, Hidefumi Suzuki, Ryota Abe, Hidenori Inohara, Yasufumi Kaneda, Hidehisa Takahashi, and Keisuke Nimura

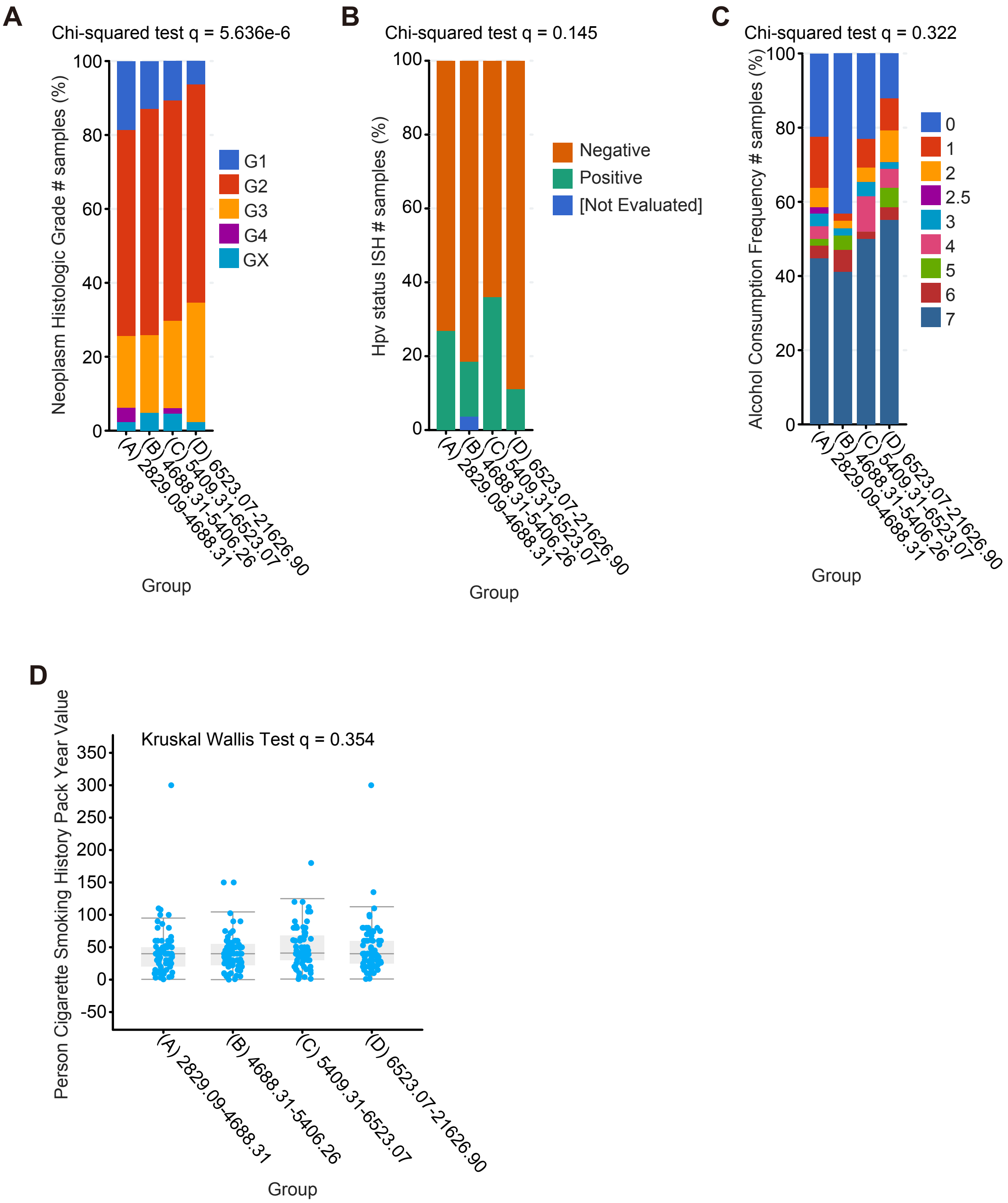

Figure S1. Correlation between SF3B2 expression and clinical information. (**A**-**C**) Cumulative bar chart of neoplasm histologic grade (**A**), HPV status (**B**), and alcohol consumption frequency (**C**). (**D**) Dot plot of person cigarette smoking history pack-year value. Clinical data were analyzed using cBioPortal.

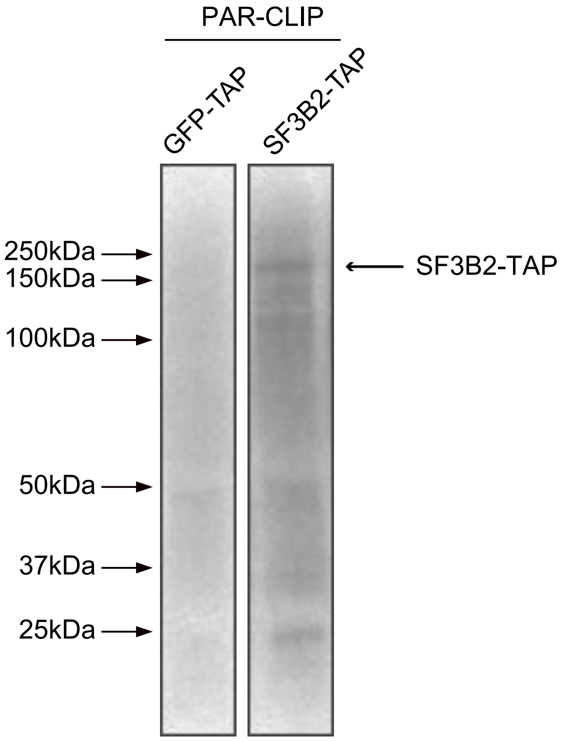

Figure S2. Autoradiograph of SF3B2 PAR-CLIP. 32P-labeled RNA crosslinking-SF3B2-TAP was detected by autoradiograph. Proteins were separated in SDS-PAGE and transferred to the membrane.

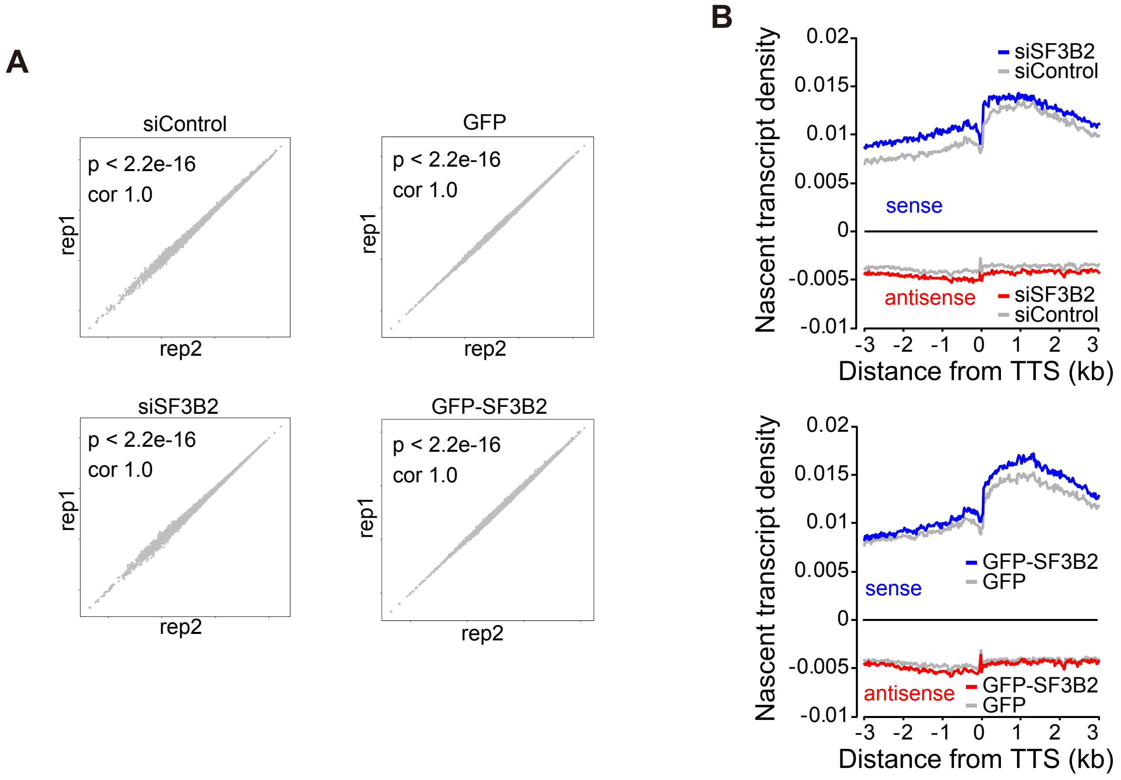

Figure S3. RNA polymerase II activity in SF3B2 knockdown or overexpression. (**A**) Dot plot of correlation between replicate 1 (rep 1) and replicate 2 (rep 2) in each PRO-seq library. (**B**) Line plot of nascent transcript density around TTS in siSF3B2 and siControl-treated FaDu cells or GFP- and GFP-SF3B2-FaDu cells.

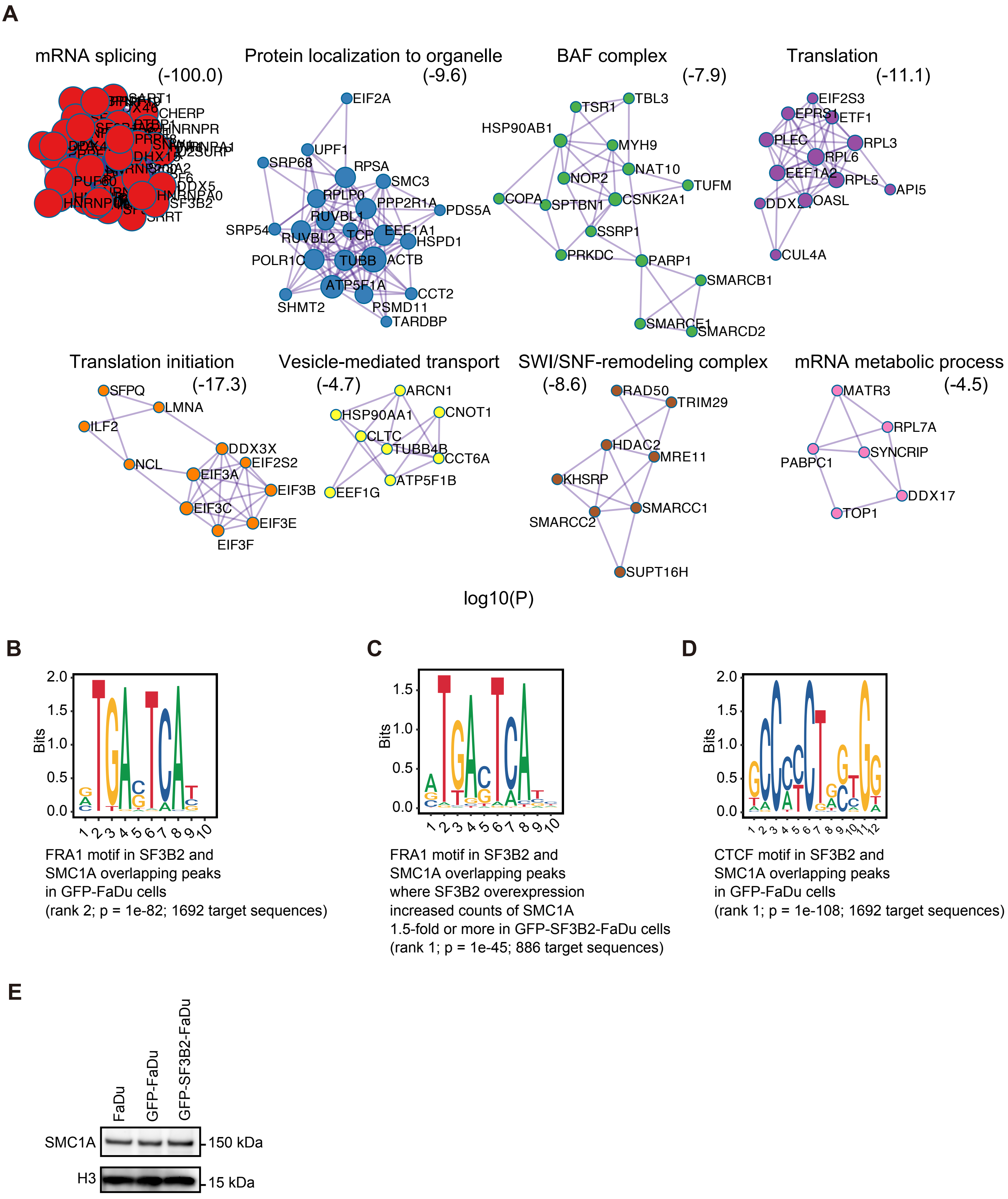

Figure S4. Protein interaction network between SF3B2-associated proteins and DNA-binding motifs. (**A**) Representative protein interaction networks are shown with p value. The network of protein interaction was analyzed using Metascape. (**B**) FRA1 motif in SF3B2 and SMC1A overlapping peaks in GFP-FaDu cells. (**C**) FRA1 motif in SF3B2 and SMC1A overlapping peaks where SF3B2 overexpression increased counts of SMC1A 1.5-fold or more. (**D**) CTCF motif in SF3B2 and SMC1A overlapping peaks in GFP-FaDu cells. (**E**) Western blots of SMC1A and H3 in wild type and GFP or GFP-SF3B2 stably-expressing FaDu cells.

Figure 1C

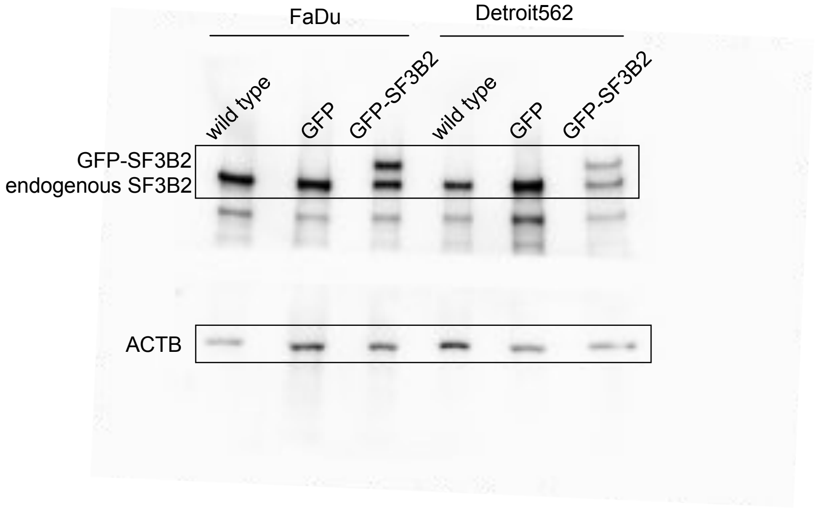

Figure 1E

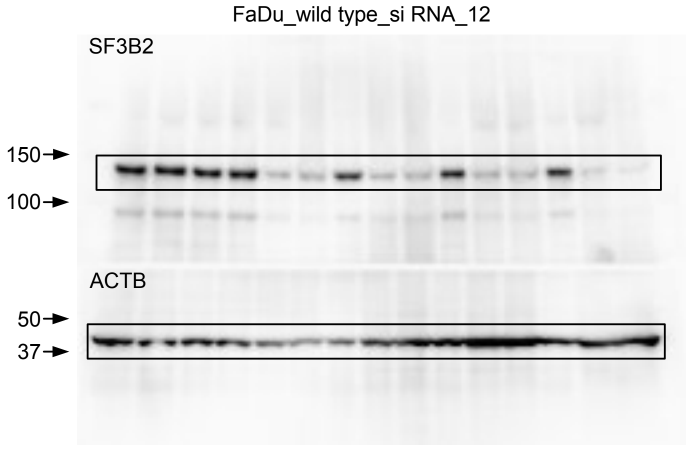

Figure 5A

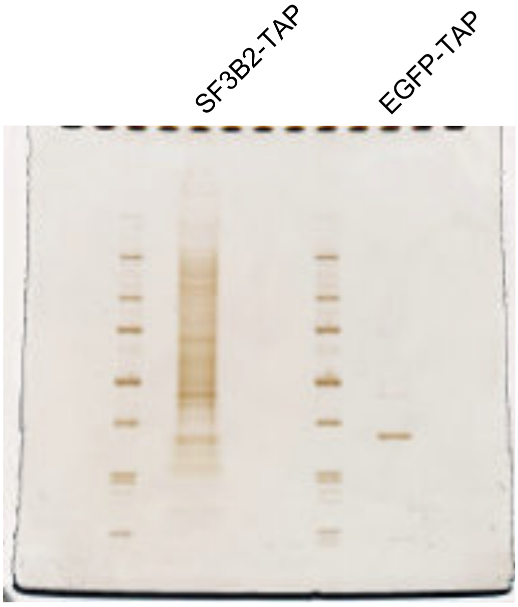

Figure 5A

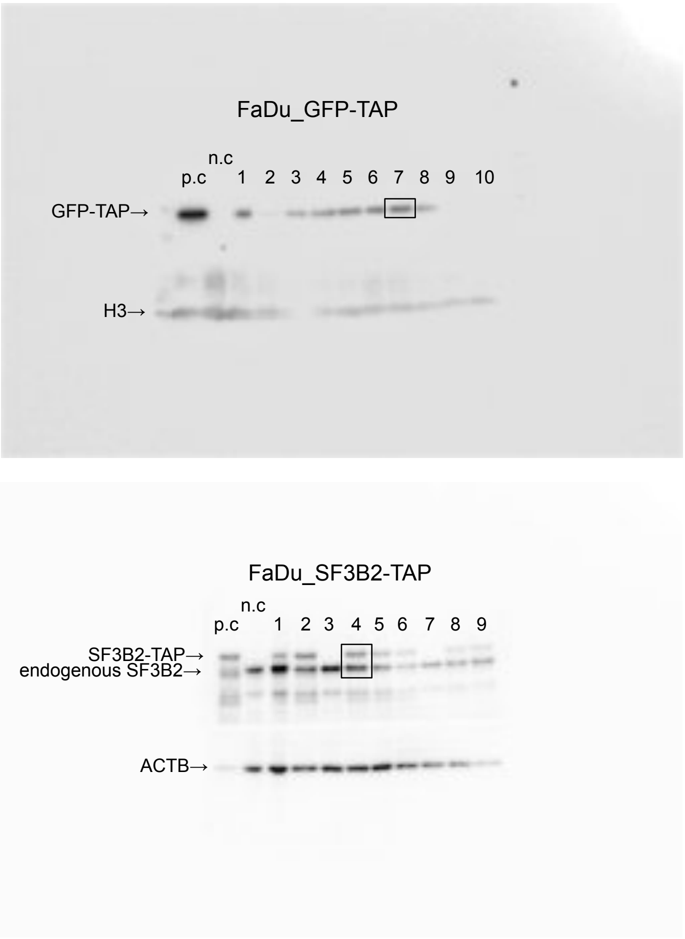

Figure S4

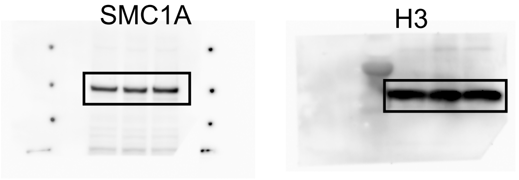

Figure S5. Original images of western blots and silver staining.

Extended Data Table 1. Correlation between replicates of sequencing libraries.

Figure	Library type	Sample 1	Sample 2	Peason correlation	p value
Figure 2	RNAseq	FaDu siSF3B2 rep1	FaDu siSF3B2 rep2	0.9962762	< 2.2e-16
	RNAseq	FaDu siSF3B2 rep1	FaDu siSF3B2 rep3	0.9938214	< 2.2e-16
	RNAseq	FaDu siSF3B2 rep2	FaDu siSF3B2 rep3	0.9964271	< 2.2e-16
	RNAseq	FaDu siControl rep1	FaDu siControl rep2	0.9972366	< 2.2e-16
	RNAseq	FaDu siControl rep1	FaDu siControl rep3	0.9972208	< 2.2e-16
	RNAseq	FaDu siControl rep2	FaDu siControl rep3	0.9972477	< 2.2e-16
	RNAseq	FaDu GFP rep1	FaDu GFP rep2	0.9966088	< 2.2e-16
	RNAseq	FaDu GFP-SF3B2 rep1	FaDu GFP-SF3B2 rep2	0.9955787	< 2.2e-16
	RNAseq	FaDu WT rep1	FaDu WT rep2	0.996255	< 2.2e-16
	RNAseq	FaDu WT rep1	FaDu WT rep3	0.9950966	< 2.2e-16
	RNAseq	FaDu WT rep2	FaDu WT rep3	0.9972965	< 2.2e-16
	CUT&TAG	FaDu SF3B2 CUT&TAG rep1	FaDu SF3B2 CUT&TAG rep2	0.9816634	< 2.2e-16
	CUT&TAG	FaDu SF3B2 CUT&TAG rep1	FaDu SF3B2 CUT&TAG rep3	0.9329757	< 2.2e-16
	CUT&TAG	FaDu SF3B2 CUT&TAG rep2	FaDu SF3B2 CUT&TAG rep3	0.9317123	< 2.2e-16
	CUT&TAG	FaDu H3 CUT&TAG rep1	FaDu H3 CUT&TAG rep2	0.9845071	< 2.2e-16
	CUT&TAG	FaDu H3 CUT&TAG rep1	FaDu H3 CUT&TAG rep3	0.9877875	< 2.2e-16
	CUT&TAG	FaDu H3 CUT&TAG rep2	FaDu H3 CUT&TAG rep3	0.9926988	< 2.2e-16
	PAR-CLIP	FaDu SF3B2 PAR-CLIP rep1	FaDu SF3B2 PAR-CLIP rep2	0.9199214	< 2.2e-16
Figure 3	PROseq	FaDu siControl ProSeq rep1	FaDu siControl ProSeq rep2	0.9982939	< 2.2e-16
	PROseq	FaDu siSF3B2 ProSeq rep1	FaDu siSF3B2 ProSeq rep2	0.9984743	< 2.2e-16
	PROseq	FaDu GFP ProSeq rep1	FaDu GFP ProSeq rep2	0.9992648	< 2.2e-16
	PROseq	FaDu GFP-SF3B2 ProSeq rep1	FaDu GFP-SF3B2 ProSeq rep2	0.9989769	< 2.2e-16
Figure 5	ChIPseq	FaDu-GFP SMC1A ChIPseq rep1	FaDu-GFP SMC1A ChIPseq rep2	0.9927736	< 2.2e-16
	ChIPseq	FaDu-GFP-SF3B2 SMC1A ChIPseq rep1	FaDu-GFP-SF3B2 SMC1A ChIPseq rep2	0.9922341	< 2.2e-16
	ChIPseq	FaDu-GFP CTCF ChIPseq rep1	FaDu-GFP CTCF ChIPseq rep2	0.9843584	< 2.2e-16
	ChIPseq	FaDu-GFP-SF3B2 CTCF ChIPseq rep1	FaDu-GFP-SF3B2 CTCF ChIPseq rep2	0.9915084	< 2.2e-16
